# Supplementary material for: GhostKnockoff inference empowers identification of putative causal variants in genome-wide association studies
Source: Nat Commun. 2022 Nov 23;13:7209. doi: 10.1038/s41467-022-34932-z (PMC9684164; doi:10.1038/s41467-022-34932-z)
Supplement: Supplementary file 1 — Supplementary Information [file 41467_2022_34932_MOESM1_ESM.pdf]

Supplementary Information for "GhostKnockoff inference empowers identification of putative causal variants in genome-wide association studies"

Zihuai He[1,2#], Linxi Liu[3], Michael E. Belloy[1], Yann Le Guen[1,4], Aaron Sossin[5], Xiaoxia Liu[1], Xinran Qi[1], Shiyang Ma[6], Prashnna K. Gyawali[1], Tony Wyss-Coray[1], Hua Tang[7], Chiara Sabatti[5], Emmanuel Candès[8,9], Michael D. Greicius[1], Iuliana Ionita-Laza[6]

[1]Department of Neurology and Neurological Sciences, Stanford University, Stanford, CA 94305, USA

[2]Quantitative Sciences Unit, Department of Medicine, Stanford University, Stanford, CA 94305, USA

[3]Department of Statistics, University of Pittsburgh, Pittsburgh, PA 15260, USA

[4]Institut du Cerveau - Paris Brain Institute - ICM, Paris, 75013, France

[5]Department of Biomedical Data Science, Stanford University, Stanford, CA 94305, USA

[6]Department of Biostatistics, Columbia University, New York, NY 10032, USA

[7]Department of Genetics, Stanford University, Stanford, CA 94305, USA

[8]Department of Statistics, Stanford University, Stanford, CA 94305, USA

[9]Department of Mathematics, Stanford University, Stanford, CA 94305, USA

# Correspondence to: Zihuai He (zihuai@stanford.edu)

Supplementary Methods

Proof of single/multiple knockoffs based inference using summary statistics

Assume the original variables have been normalized to have mean 0 and variance 1. For single knockoff, given individual-level data, we generate knockoffs $\widetilde{\boldsymbol{G}}$ by the conditional distribution given the original genetic variants:

$$\widetilde{\boldsymbol{G}}_i|\boldsymbol{G}_i \sim \boldsymbol{N}(\boldsymbol{G}_i\boldsymbol{P}^{\boldsymbol{T}}, \boldsymbol{V}), \qquad (1)$$

$$\boldsymbol{P} = (\boldsymbol{I} - \boldsymbol{D}\boldsymbol{\Sigma}^{-\mathbf{1}}), \qquad \boldsymbol{V} = 2\boldsymbol{D} - \boldsymbol{D}\boldsymbol{\Sigma}^{-\mathbf{1}}\boldsymbol{D}, \qquad (2)$$

where $\boldsymbol{I}$ is a $p \times p$ identity matrix; $\boldsymbol{\Sigma}$ is the correlation matrix of $\boldsymbol{G}_i$ that characterizes the linkage disequilibrium; $\boldsymbol{D} = diag(s_1, \dots, s_p)$ is a diagonal matrix given by solving the following convex optimization problem:

$$minimize \sum_j |1 - s_j|, subject\ to \begin{cases} 2\boldsymbol{\Sigma} - \boldsymbol{D} \succcurlyeq 0, \\ s_j \ge 0,\ \ 1 \le j \le p. \end{cases} (3)$$

The per-sample score statistic (original and knockoff) can be written as

$$\boldsymbol{G}_i^{\boldsymbol{T}} Y_i, \qquad \widetilde{\boldsymbol{G}}_i^{\boldsymbol{T}} Y_i \coloneqq \boldsymbol{P}\boldsymbol{G}_i^{\boldsymbol{T}} Y_i + \boldsymbol{\phi}_i^{\boldsymbol{T}} Y_i, \qquad (4)$$

where $\boldsymbol{\phi}_i = (\phi_{i1}, \dots, \phi_{ip}) \sim N(0, \boldsymbol{V})$ is a vector of random variables that follows a multivariate normal distribution with mean 0 covariance $\boldsymbol{V}$. The score test statistic for original and knockoff variables are

$$\boldsymbol{S} = \boldsymbol{G}^{\boldsymbol{T}}\boldsymbol{Y}, \qquad \widetilde{\boldsymbol{S}} = \widetilde{\boldsymbol{G}}^{\boldsymbol{T}}\boldsymbol{Y} = \boldsymbol{P}\boldsymbol{G}^{\boldsymbol{T}}\boldsymbol{Y} + \boldsymbol{\Phi}^{\boldsymbol{T}}\boldsymbol{Y}, \qquad (5)$$

where $\boldsymbol{G}$, $\widetilde{\boldsymbol{G}}$, $\boldsymbol{\eta}$, $\boldsymbol{\Phi}$ are all $n \times p$ matrix stacking corresponding per-sample vectors; $\boldsymbol{Y} = (Y_1, \dots, Y_n)^T$. Since the rows of $\boldsymbol{\Phi}$ follows i.i.d multivariate normal distribution $\boldsymbol{N}(\boldsymbol{0}, \boldsymbol{V})$, equivalently, we can write

$$\widetilde{\boldsymbol{S}} = \boldsymbol{P}\boldsymbol{S} + \sqrt{\sum\nolimits_{\boldsymbol{i}=\mathbf{1}}^{\boldsymbol{n}} Y_i^2} \cdot \boldsymbol{E} \qquad (6)$$

where $\boldsymbol{E}$ is a random vector following multivariate normal distribution $\boldsymbol{N}(\boldsymbol{0}, \boldsymbol{V})$. Then

$$\widetilde{\boldsymbol{Z}}_{\boldsymbol{score}}|\boldsymbol{G}, \boldsymbol{Y} \sim \boldsymbol{N}\left(\boldsymbol{P}\boldsymbol{Z}_{\boldsymbol{score}}, \frac{\sum_{\boldsymbol{i}=\mathbf{1}}^{\boldsymbol{n}} Y_i^2}{n} \cdot \boldsymbol{V}\right). \ (7)$$

Assuming Y_i has been centered at the conditional mean given the covariates and scaled with variance 1, under the null hypothesis (H_0: $\boldsymbol{\beta} = 0$) as $n \to \infty$

$$\frac{\sum_{\boldsymbol{i}=\mathbf{1}}^{\boldsymbol{n}} Y_i^2}{n} \to 1 \text{ in probablity.} \ (8)$$

Therefore, asymptotically, we can directly generate the score statistic for knockoff variables as

$$\widetilde{\boldsymbol{Z}}_{\boldsymbol{score}}|\boldsymbol{G}, \boldsymbol{Y} \sim \boldsymbol{N}(\boldsymbol{P}\boldsymbol{Z}_{\boldsymbol{score}}, \boldsymbol{V}). \ (9)$$

For multiple knockoffs, we can generate multiple knockoffs $\widetilde{\boldsymbol{G}} = \left(\widetilde{\boldsymbol{G}}^{(\mathbf{1})}, \dots, \widetilde{\boldsymbol{G}}^{(\boldsymbol{M})}\right)$ by the conditional distribution given the original genetic variants:

$$\widetilde{\boldsymbol{G}}_i|\boldsymbol{G}_i \sim \boldsymbol{N}(\boldsymbol{G}_i\boldsymbol{P}^{\boldsymbol{T}}, \boldsymbol{V}), \qquad (10)$$

$$\boldsymbol{P}=\begin{pmatrix}\boldsymbol{I}-\boldsymbol{D}\boldsymbol{\Sigma}^{-1}\\ \dots\\ \boldsymbol{I}-\boldsymbol{D}\boldsymbol{\Sigma}^{-1}\end{pmatrix},\qquad \boldsymbol{V}=\begin{pmatrix}\boldsymbol{C} & \boldsymbol{C}-\boldsymbol{D} & \dots & \boldsymbol{C}-\boldsymbol{D}\\ \boldsymbol{C}-\boldsymbol{D} & \boldsymbol{C} & \dots & \boldsymbol{C}-\boldsymbol{D}\\ \dots & \dots & \dots & \dots\\ \boldsymbol{C}-\boldsymbol{D} & \boldsymbol{C}-\boldsymbol{D} & \dots & \boldsymbol{C}\end{pmatrix},\qquad (11)$$

where $\boldsymbol{I}$ is a $p \times p$ identity matrix; $\boldsymbol{\Sigma}$ is the correlation matrix of $\boldsymbol{G_i}$ that characterizes the linkage disequilibrium; $\boldsymbol{C} = 2\boldsymbol{D} - \boldsymbol{D}\boldsymbol{\Sigma}^{-\boldsymbol{1}}\boldsymbol{D}$; $\boldsymbol{D} = \mathrm{diag}(s_1, \dots, s_p)$ is a diagonal matrix given by solving the following convex optimization problem:

$$\text{minimize} \sum_j \left|1 - s_j\right|, \text{subject to} \begin{cases}\dfrac{M+1}{M}\boldsymbol{\Sigma} - \boldsymbol{D} \succcurlyeq 0\\ s_j \geq 0,\;\; 1 \leq j \leq p\end{cases} \quad (12)$$

Following similar derivations as for single knockoff, the score test statistic can be written as

$$\boldsymbol{S} = \boldsymbol{G}^{\boldsymbol{T}}\boldsymbol{Y},\qquad \tilde{\boldsymbol{S}} = \tilde{\boldsymbol{G}}^{\boldsymbol{T}}\boldsymbol{Y}|\boldsymbol{G},\boldsymbol{Y} \sim \boldsymbol{N}(\boldsymbol{PS}, \left(\sum_{\boldsymbol{i=1}}^{\boldsymbol{n}} Y_i^2\right) \cdot \boldsymbol{V}),\qquad (13)$$

where now $\tilde{\boldsymbol{S}} = \left(\tilde{\boldsymbol{S}}^{(\boldsymbol{1})\boldsymbol{T}}, \dots, \tilde{\boldsymbol{S}}^{(\boldsymbol{M})\boldsymbol{T}}\right)^{\boldsymbol{T}}$ is a $p \times M$ dimensional vector of knockoff score test statistics. Asymptotically, we can still directly generate the test statistic for knockoff variables by

$$\boldsymbol{Z_{score}} = \frac{1}{\sqrt{n}}\boldsymbol{S},\qquad \tilde{\boldsymbol{Z}}_{\boldsymbol{score}}|\boldsymbol{G},\boldsymbol{Y} \sim \boldsymbol{N}(\boldsymbol{P}\boldsymbol{Z_{score}}, \boldsymbol{V}).\ (14)$$

The above derivation is based on hypothetically constructing model-X knockoffs. The advantage of the model-X framework mainly lies in the following two perspectives: first, it does not impose any constraints on the dimension, implying that the method can still provide valid inference even when the dimension is much larger than the sample size, which is particularly useful for analysis of GWAS/whole genome sequencing data; second, it does not make any assumption on the model for the conditional distribution of the outcome given genetic variables, i.e., the method can be applied to both continuous and binary traits.

Supplementary Notes

Consistency between the estimated study correlations and similarities in the design of the considered Alzheimer's disease studies

The estimated study correlations $cor.S_{ij}$ (**Figure 4A**) are consistent with our knowledge of overlap and other factors, such as differences in phenotype definition, analysis strategies (e.g. statistical model), and quality control, that can affect the correlations between these studies. For example, Kunkle et al. (2019) and Schwartzentruber et al. (2021) are highly correlated partly because the latter study is a meta-analysis that includes summary statistics from Kunkle et al. (2019). The three WES studies (Bis et al. (2019), Le Guen et al. (2021) and our in-house ADSP whole-exome sequencing analysis) are all based on the ADSP cohorts with different preprocessing steps, therefore they appear highly correlated to each other. Some weaker correlations are observed for studies that use different phenotype definitions. For example, Huang et al. (2017) is weakly correlated with other major AD GWAS because the authors performed a time-to-event survival analysis; the correlation between Le Guen et al. (2021) and Bis et al. (2019) is weaker than that between our in-house ADSP WES analysis and Bis et al. (2019), because Le Guen et al. (2021) used a new age-informed AD phenotype instead of clinical AD.

Supplementary Figures

Supplementary Figure 1: Empirical simulation studies for power and FDR to compare different knockoff generators. Two cohorts are randomly sampled from the same population. The panels show power and FDR based on 1000 replicates for different types of traits (quantitative and dichotomous) and different levels of sample overlap (0%/25%/50%), with different target FDR varying from 0 to 0.2. All methods are based on single knockoff for a fair comparison. GhostKnockoff-S: the proposed single knockoff method based on the meta-analysis of Z-scores calculated separately from each individual cohort. SummaryStat knockoff-S: the proposed single knockoff method based on Z-scores from the pooled data; HMM/SCIP/SecondOrder knockoff-S: existing knockoff generators based on individual level data; We additionally present HMM knockoff+Lasso-S, which corresponds to the KnockoffZoom method proposed by Sesia et al. (2020) at single variant resolution.

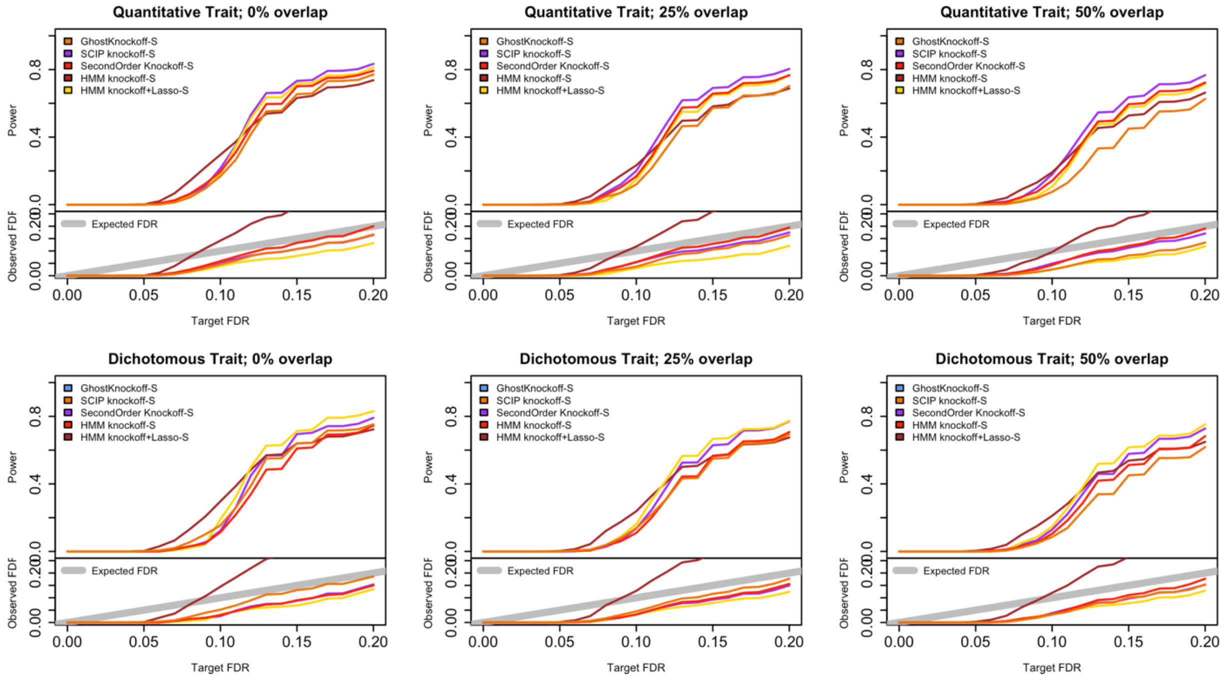

Supplementary Figure 2: Empirical simulation studies for power and FDR in the presence of study specific variants. Two cohorts are randomly sampled from the same population with 25% sample overlap. The panels show power and FDR based on 1000 replicates for different types of traits (quantitative and dichotomous), and different levels of unobserved variants per study (0%/10%/20%), with different target FDR varying from 0 to 0.2. GhostKnockoff-M/S: the proposed multiple/single knockoff method based on the meta-analysis of Z-scores calculated separately from each individual cohort. IndividualData Knockoff-M/S: the multiple/single knockoff inference based on applying SecondOrder knockoff generator to pooled individual level data. All methods are based on the same definition of feature importance score.

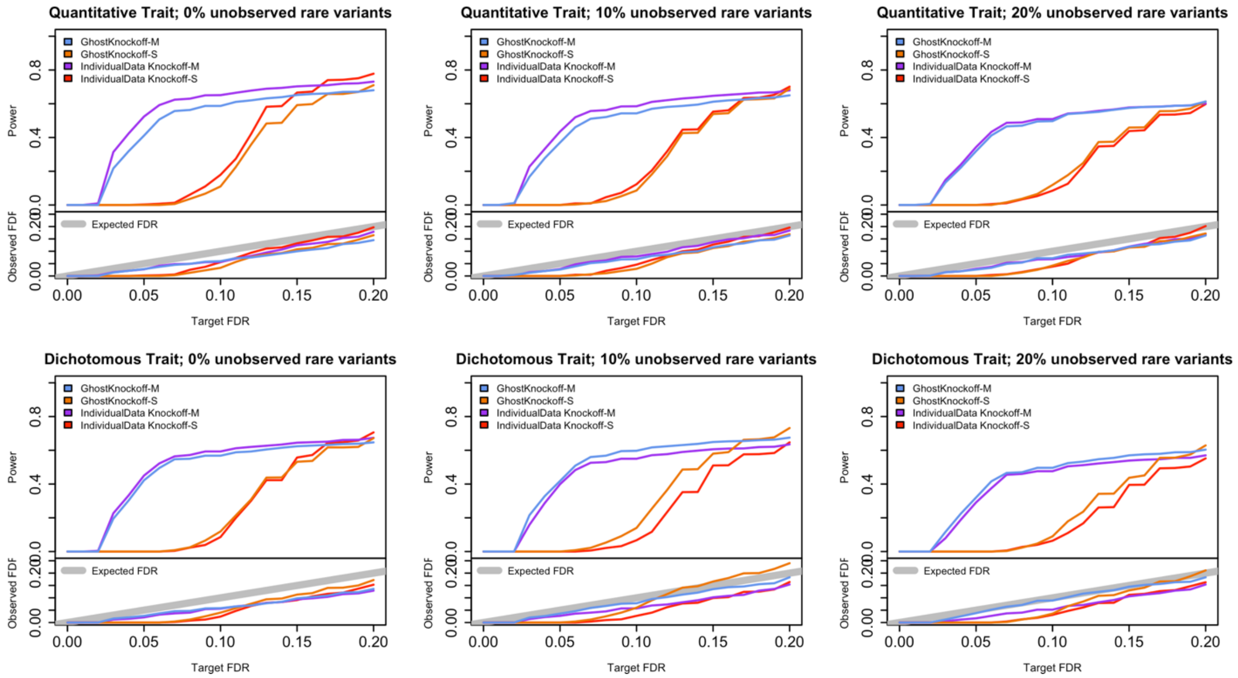

Supplementary Figure 3: Replication of variants and loci identified by GhostKnockoff in larger studies. The analysis reflects the application of the proposed method to a subset of samples and the validation of the findings when we increase the sample size. We present the Manhattan plot of W statistics (truncated at 100 for clear visualization) from GhostKnockoff with target FDR at 0.05 (red dotted line; loci are highlighted in red) and 0.10 (blue dotted line; loci are highlighted in blue). **A.** GhostKnockoff analysis based on summary statistics from Kunkle et al. (2019). **B.** GhostKnockoff analysis based on summary statistics from Schwartzentruber et al. (2021), a study aggregating samples from Kunkle et al. (2019) and UK Biobank based on a proxy AD phenotype. **C.** The proposed GhostKnockoff meta-analysis based on the optimal weights combining nine studies. Variant density is shown at the bottom of Manhattan plot (number of variants per 1Mb).

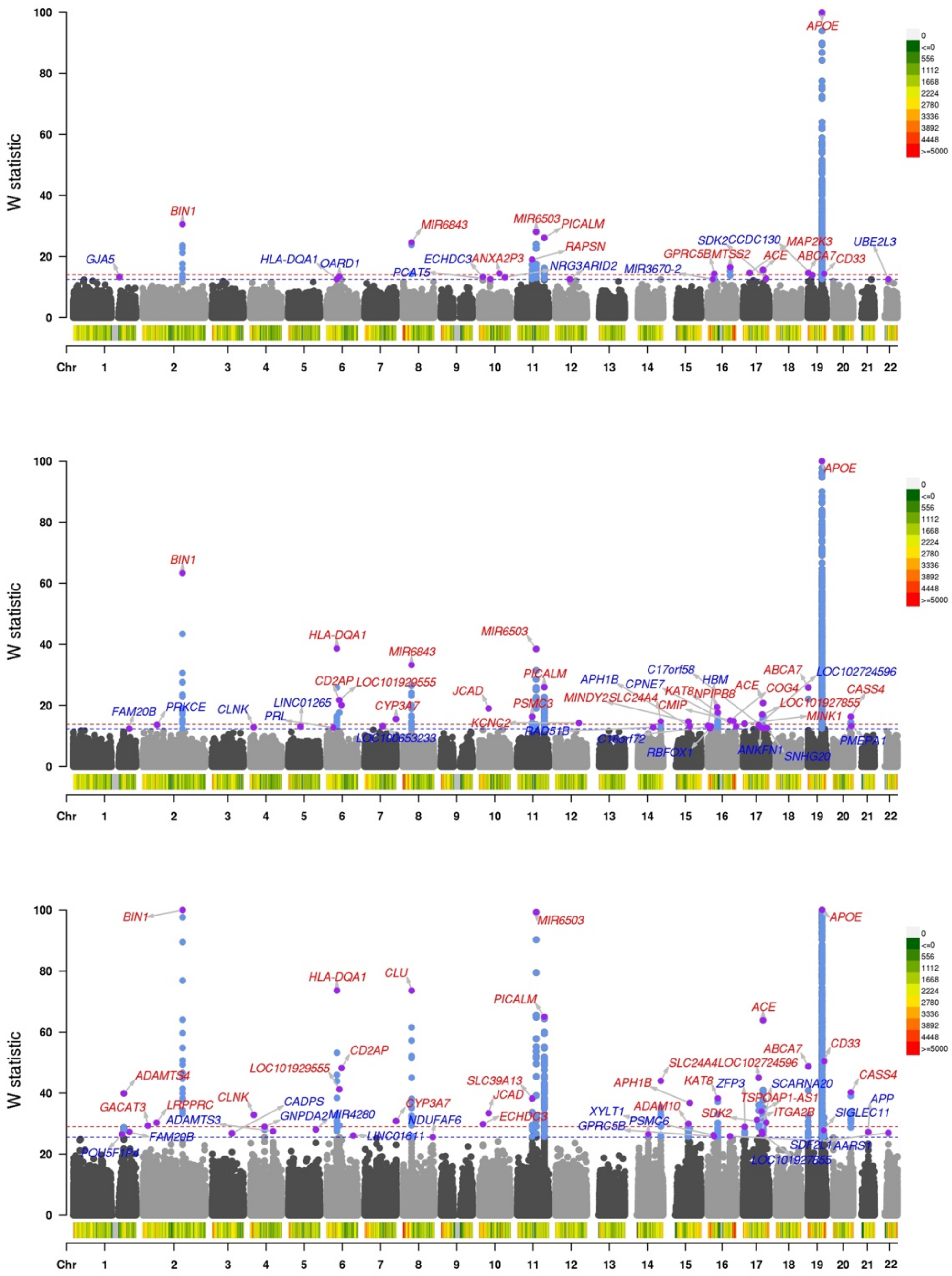

Supplementary Figure 4: Comparison between sample size weights (top panel; 29 loci at FDR 0.05; 46 loci at FDR 0.10) vs. optimal weights (bottom panel; 34 loci at FDR 0.05; 50 loci at FDR 0.10). We present the Manhattan plot of W statistics (truncated at 100 for clear visualization) from GhostKnockoff with target FDR at 0.05 (red) and 0.10 (blue). Variant density is shown at the bottom of Manhattan plot (number of variants per 1Mb).

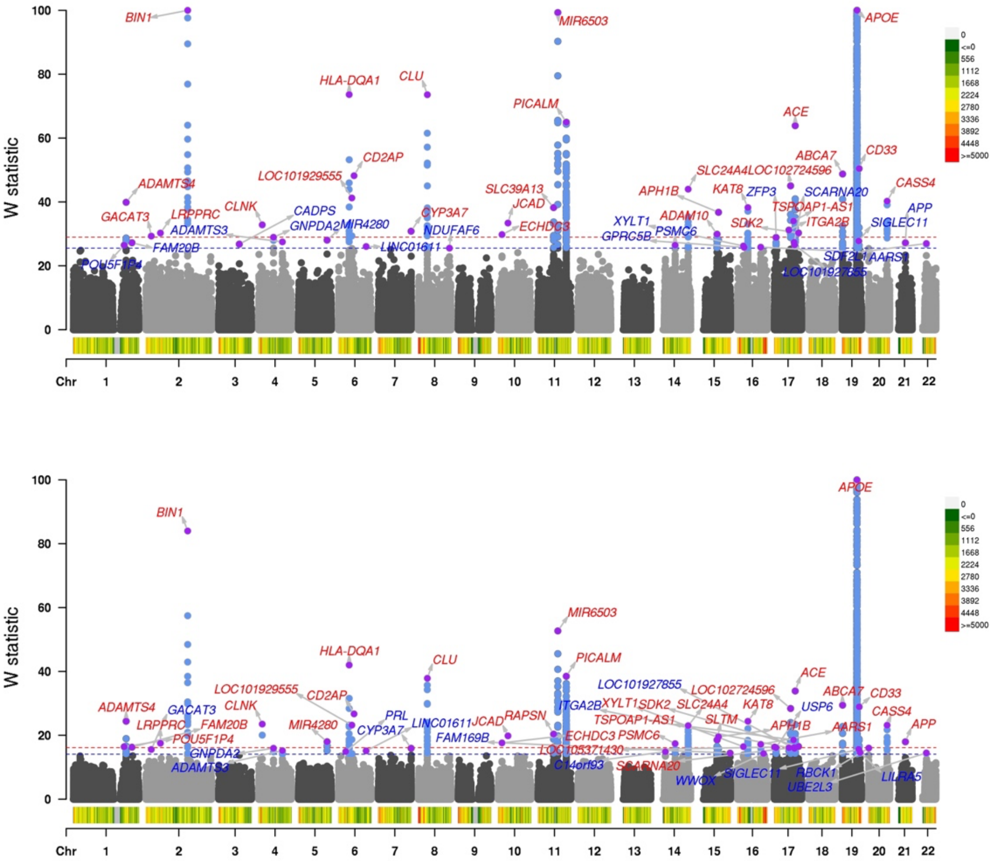

Supplementary Figure 5: Functional enrichment analysis. Functional scores of variants identified by different methods are compared using one-sided t-tests. Overlap: GWAS discoveries that overlap with knockoff inference.

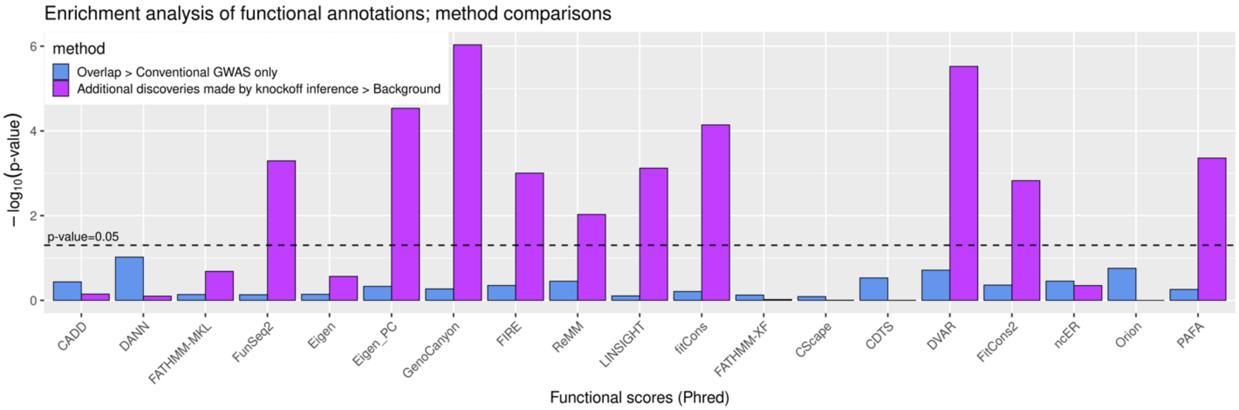

Supplementary Tables

Supplementary Table 1. Replication of variants identified by GhostKnockoff in larger studies. The analysis reflects the application of the proposed method to a subset of samples and the validation of the findings when we increase the sample size. We present the number of identified variants by applying GhostKnockoff to summary statistics from Kunkle et al. (2019), Schwartzentruber et al. (2021) (a study aggregating samples from Kunkle et al. (2019) and UK Biobank based on a proxy AD phenotype), and the proposed GhostKnockoff meta-analysis based on the optimal weights combining nine studies. A genetic variant is replicated if the same variant is also identified in the next larger study with a smaller p-value and the same direction of effect.

		Source of summary statistics		
		Kunkle et al. (2019)	Schwartzentruber et al. (2021)	Proposed meta-analysis of all nine studies
FDR=0.05	Total number of discoveries	385	634	764
	Number (proportion) of replicated discoveries	338 (87.8%)	447 (70.5%)	-
FDR=0.10	Total number of discoveries	448	724	935
	Number (proportion) of replicated discoveries	370 (82.6%)	510 (70.4%)	-

FDR: False discovery rate.

Supplementary Table 2. Loci associated with Alzheimer's disease at FDR=0.05. For each locus, we present the representative variant with the largest W-statistic and the nearest gene within +-1Mb. The physical positions of each variant are given in build hg38.

Variant	Proximal Gene	MAF	q	Jansen et al.	Kunkle et al.	Schwartzentruber et al.	Bis et al. WES	In-house ADGC	In-house ADSP WES	In-house ADSP WGS	LeGuan et al. WES	Huang et al.	Direction of effects	scRNAseq DEG minP
1:155419060:A:T	POU5F1P4	0.024	0.0448	1.1E-04	3.3E-03	2.2E-04	NA	4.9E-01	NA	6.8E-01	NA	1.1E-02	+++0+0+0+	NA
1:161185602:G:A	ADAMTS4	0.242	0.0035	1.9E-10	2.4E-02	4.3E-08	NA	7.9E-02	NA	1.5E-02	NA	3.3E-01	+++0+0+0+	6.60E-55
1:178992361:C:T	FAM20B	0.028	0.0477	1.1E-03	4.0E-02	4.0E-05	NA	7.1E-01	NA	9.8E-01	NA	3.8E-02	+++0-0+0+	2.00E-01
2:44026309:T:C	LRPPRC	0.026	0.0302	1.4E-04	6.0E-02	3.9E-04	NA	2.4E-02	NA	2.7E-04	NA	2.2E-01	---0-0-0-	9.60E-05
2:127135234:C:T	BIN1	0.38	0.0007	1.3E-29	4.1E-28	1.1E-54	NA	2.2E-27	NA	3.9E-11	NA	4.5E-07	+++0+0+0+	6.20E-08
4:11026080:T:C	CLNK	0.282	0.0056	4.2E-09	5.7E-05	8.1E-11	NA	4.2E-05	NA	3.2E-02	NA	8.5E-03	---0-0-0-	3.70E-01
5:87002714:C:T	MIR4280	0.191	0.0250	2.1E-06	1.7E-02	4.5E-05	NA	1.7E-06	NA	9.4E-03	NA	3.7E-03	+++0+0+0+	NA
6:32637301:A:G	HLA-DQA1	0.05	0.0007	1.1E-09	2.7E-05	2.5E-14	NA	NA	NA	NA	NA	2.0E-01	---00000+	1.70E-01
6:40783137:A:T	LOC101929555	0.014	0.0059	3.7E-05	1.4E-05	4.4E-08	NA	3.0E-03	NA	4.3E-02	NA	NA	0	NA
6:47479305:T:A	CD2AP	0.251	0.0027	9.2E-09	1.9E-07	1.0E-09	NA	1.5E-07	NA	2.3E-02	NA	6.0E-02	+++0+0+0+	1.00E-04
8:27598736:T:C	CLU	0.405	0.0012	1.1E-17	6.0E-16	2.9E-24	NA	9.0E-07	NA	1.4E-01	NA	1.8E-04	+++0+0+0+	1.20E-07
10:11678621:C:T	ECHDC3	0.349	0.0293	7.5E-08	8.7E-06	1.6E-10	NA	4.2E-08	NA	1.3E-02	NA	4.8E-04	+++0+0+0+	2.70E-01
10:29966853:G:A	JCAD	0.016	0.0154	2.0E-04	4.1E-02	5.2E-06	NA	1.9E-01	NA	6.3E-01	NA	NA	0	1.60E-06
11:47440232:A:G	RAPSN	0.374	0.0134	1.8E-06	1.2E-07	1.1E-09	NA	1.0E-02	NA	2.9E-02	NA	1.4E-02	+++0+0+0+	1.70E-01
11:60212842:C:G	MIR6503	0.401	0.0007	2.1E-13	3.0E-15	5.6E-18	NA	2.4E-09	NA	1.7E-03	NA	4.1E-02	---0-0-0-	NA
11:86089237:G:A	PICALM	0.349	0.0012	2.1E-17	1.0E-14	2.5E-25	NA	7.0E-08	NA	2.7E-03	NA	4.5E-11	+++0+0+0+	1.90E-07
14:52710264:A:C	PSMC6	0.114	0.0321	2.0E-05	2.6E-04	1.4E-08	NA	2.4E-02	NA	NA	NA	2.0E-03	+++0+000+	1.20E-03
14:92469490:G:A	SLC24A4	0.23	0.0062	2.2E-09	1.4E-06	5.4E-10	NA	2.6E-03	NA	3.5E-03	NA	2.4E-02	---0-0-0-	1.50E-02
15:58889786:G:A	SLTM	0.255	0.0209	2.7E-07	1.0E-02	7.3E-06	NA	3.3E-03	NA	1.2E-01	NA	3.9E-03	+++0+0+0+	7.10E-04
15:63277703:C:T	APH1B	0.135	0.0159	3.4E-08	2.4E-04	1.1E-08	1.4E-02	9.9E-03	3.3E-01	4.0E-02	1.1E-01	2.3E-01	+++++++++	1.90E-02
16:17478817:T:C	XYLT1	0.221	0.0444	7.8E-04	1.3E-02	5.8E-04	NA	6.2E-01	NA	6.5E-01	NA	3.7E-03	+++0+0+0+	9.50E-06
16:31121341:G:A	KAT8	0.296	0.0035	3.8E-08	7.6E-03	6.3E-09	NA	4.1E-03	NA	1.3E-01	NA	2.0E-02	---0-0-0-	1.60E-01
16:70258841:T:C	AARS1	0.203	0.0331	1.8E-03	4.6E-03	7.3E-04	NA	3.5E-01	NA	7.3E-01	NA	7.5E-04	---0-0+0-	NA
17:388402:T:C	LOC105371430	0.42	0.0471	1.2E-03	1.3E-01	1.5E-04	NA	5.5E-01	NA	9.1E-01	NA	1.2E-02	+++0+0+0+	NA

Supplementary Table 2 (continue). Loci associated with Alzheimer's disease at FDR=0.05. For each locus, we present the representative variant with the largest W-statistic and the nearest gene within +-1Mb. The physical positions of each variant are given in build hg38.

Variant	Proximal Gene	MAF	q	Jansen et al.	Kunkle et al.	Schwartzentruber et al.	Bis et al. WES	In-house ADGC	In-house ADSP WES	In-house ADSP WGS	LeGuan et al. WES	Huang et al.	Direction of effects	scRNAseq DEG minP
17:49391824:G:A	LOC102724596	0.427	0.0025	1.7E-07	1.0E-02	5.1E-05	NA	4.2E-02	NA	9.3E-01	NA	2.5E-02	---0-0+0-	NA
17:58320645:C:G	TSPOAP1-AS1	0.448	0.0209	2.6E-08	8.5E-06	3.3E-05	NA	2.0E-03	NA	6.6E-02	NA	6.0E-01	---0-0-0-	1.3E-02
17:60239372:C:T	SCARNA20	0.199	0.0492	1.3E-03	4.4E-03	2.1E-03	NA	9.7E-01	NA	1.7E-01	NA	8.8E-03	+++0-0+0+	NA
17:63482562:C:T	ACE	0.388	0.0017	3.9E-07	3.9E-04	1.5E-07	5.4E-02	8.7E-05	3.7E-02	1.4E-02	7.5E-03	6.3E-03	+++++++++	1.2E-02
17:73384739:C:T	SDK2	0.204	0.0420	8.5E-04	1.4E-03	1.7E-04	NA	3.1E-02	NA	9.5E-01	NA	8.0E-02	---0-0+0-	6.4E-02
19:1046077:C:T	ABCA7	0.116	0.0025	2.6E-07	1.4E-03	7.9E-11	NA	3.9E-03	NA	5.1E-02	NA	6.3E-01	+++0+0+0+	1.1E-02
19:44908684:T:C	APOE	0.154	0.0007	0.0E+00	0.0E+00	0.0E+00	NA	NA	NA	NA	NA	2.6E-131	+++00000+	2.0E-16
19:51224706:C:A	CD33	0.31	0.0025	5.2E-09	3.6E-07	1.3E-08	NA	1.7E-04	NA	7.1E-05	NA	6.8E-04	---0-0-0-	2.0E-01
20:56443204:T:C	CASS4	0.08	0.0059	2.6E-08	9.3E-06	1.8E-08	NA	1.1E-06	NA	1.9E-02	NA	8.6E-04	---0-0-0-	1.3E-14
21:26161943:T:C	APP	0.371	0.0252	4.2E-07	1.2E-03	1.0E-07	NA	6.3E-04	NA	NA	NA	1.1E-01	+++0+000+	3.2E-16

WES: whole genome sequencing; WGS: whole genome sequencing; ADGC: Alzheimer's Disease Genetics Consortium; ADSP: Alzheimer's Disease Sequencing Project; DEG: differentially expression gene; minP; minimum p-value.

Supplementary Table 3. Accession IDs for the cohorts included in the in-house genome-wide associations study imputed using the TOPMed reference panels.

Cohort/Project	Sample count	Data Repository
A4	3465	LONI A4
ACT	2790	NIAGADS (NG00034) / dbGaP (phs000234)
ADC1	2731	NIAGADS (NG00022) / NACC
ADC2	928	NIAGADS (NG00023) / NACC
ADC3	1526	NIAGADS (NG00024) / NACC
ADC4	1054	NIAGADS (NG00068) / NACC
ADC5	1224	NIAGADS (NG00069) / NACC
ADC6	1333	NIAGADS (NG00070) / NACC
ADC7	1462	NIAGADS (NG00071) / NACC
ADDNEUROMED	315	Synapse AddNeuroMed (syn4907804)
	329	Synapse AddNeuroMed (syn4907804)
ADNI	757	LONI ADNI
	361	LONI ADNI
	327	LONI ADNI
	812	LONI ADNI
	812	LONI ADNI
ADNI-DOD	204	LONI ADNIDOD
ADGC Exome-Arrays	5180	NIAGADS (NG00081) / NACC
	1923	NIAGADS (NG00080) / NACC
	5998	NIAGADS (NG00079) / NACC
	868	NIAGADS (NG00085) / NACC
ADSP WES	20503	NIAGADS DSS (NG00067.v5) / NACC
ADSP WGS	16906	NIAGADS DSS (NG00067.v5) / NACC
Indianapolis African-American	1175	NIAGADS (NG00047)
Indianapolis Yoruba	1264	dbGaP (phs000378)
CIDR	3101	NIAGADS (NG00015) / dbGAP (phs000496)
GenADA	1571	dbGaP (phs000219)

HBTRC	338	Synapse AMP-AD (syn3159435)
	402	Synapse AMP-AD (syn3159435)
LATC	63	RADC Rush (contact:Gregory_Klein@rush.edu)
NIA-LOAD	5220	NIAGADS (NG00020)
MARS	708	RADC Rush (contact:Gregory_Klein@rush.edu)
MAYO	2099	Synapse AMP-AD (syn5591675) / NIAGADS (NG00029)
	349	Synapse AMP-AD (syn22264775)
MAYO2	314	Synapse AMP-AD (syn5550404)
	349	Synapse AMP-AD (syn22264775)
MIRAGE	397	NIAGADS (NG00031)
	1105	NIAGADS (NG00031)
MSBB	349	Synapse AMP-AD (syn3159438, syn22264775)
MTC	542	NIAGADS (NG00096)
OHSU	647	NIAGADS (NG00017)
ROSMAP	1126	RADC Rush (contact:Gregory_Klein@rush.edu) / Synapse AMP-AD
	582	RADC Rush (contact:Gregory_Klein@rush.edu) / Synapse AMP-AD
	382	RADC Rush (contact:Gregory_Klein@rush.edu) / Synapse AMP-AD
	494	RADC Rush (contact:Gregory_Klein@rush.edu)
	1196	RADC Rush (contact:Gregory_Klein@rush.edu) / Synapse AMP-AD
TARCC	625	NIAGADS (NG00097)
	2718	TARCC (contact: Bruce.Jones@UTSouthwestern.edu)
TGEN2	1599	NIAGADS (NG00028)
UPITT	2440	NIAGADS (NG00026)
UM/VU/MSSM	1153	NIAGADS (NG00042)
	864	NIAGADS (NG00042)
	445	NIAGADS (NG00042)
WASHU	670	NIAGADS (NG00030)
WASHU2	235	NIAGADS (NG00087)
WHICAP	647	NIAGADS (NG00093)
