# Supplementary material for: GhostKnockoff inference empowers identification of putative causal variants in genome-wide association studies
Source: Nat Commun. 2022 Nov 23;13:7209. doi: 10.1038/s41467-022-34932-z (PMC9684164; doi:10.1038/s41467-022-34932-z)
Supplement: Supplementary file 3 — Reporting Summary [file 41467_2022_34932_MOESM3_ESM.pdf]

nature portfolio

Corresponding author(s): Zihuai He

Last updated by author(s): Nov 2, 2022

Reporting Summary

Nature Portfolio wishes to improve the reproducibility of the work that we publish. This form provides structure for consistency and transparency in reporting. For further information on Nature Portfolio policies, see our Editorial Policies and the Editorial Policy Checklist.

Statistics

For all statistical analyses, confirm that the following items are present in the figure legend, table legend, main text, or Methods section.

n/a	Confirmed	
☐	☒	The exact sample size (*n*) for each experimental group/condition, given as a discrete number and unit of measurement
☐	☒	A statement on whether measurements were taken from distinct samples or whether the same sample was measured repeatedly
☐	☒	The statistical test(s) used AND whether they are one- or two-sided<br>*Only common tests should be described solely by name; describe more complex techniques in the Methods section.*
☐	☒	A description of all covariates tested
☐	☒	A description of any assumptions or corrections, such as tests of normality and adjustment for multiple comparisons
☐	☒	A full description of the statistical parameters including central tendency (e.g. means) or other basic estimates (e.g. regression coefficient) AND variation (e.g. standard deviation) or associated estimates of uncertainty (e.g. confidence intervals)
☐	☒	For null hypothesis testing, the test statistic (e.g. *F*, *t*, *r*) with confidence intervals, effect sizes, degrees of freedom and *P* value noted<br>*Give P values as exact values whenever suitable.*
☒	☐	For Bayesian analysis, information on the choice of priors and Markov chain Monte Carlo settings
☒	☐	For hierarchical and complex designs, identification of the appropriate level for tests and full reporting of outcomes
☐	☒	Estimates of effect sizes (e.g. Cohen's *d*, Pearson's *r*), indicating how they were calculated

Our web collection on statistics for biologists contains articles on many of the points above.

Software and code

Policy information about availability of computer code

Data collection	No software was used.
Data analysis	We have implemented GhostKnockoff in a computationally efficient R package that can be accessed at https://cran.r-project.org/web/packages/GhostKnockoff/.

For manuscripts utilizing custom algorithms or software that are central to the research but not yet described in published literature, software must be made available to editors and reviewers. We strongly encourage code deposition in a community repository (e.g. GitHub). See the Nature Portfolio guidelines for submitting code & software for further information.

Data

Policy information about availability of data

All manuscripts must include a data availability statement. This statement should provide the following information, where applicable:

- Accession codes, unique identifiers, or web links for publicly available datasets
- A description of any restrictions on data availability
- For clinical datasets or third party data, please ensure that the statement adheres to our policy

The manuscript used summary statistics from existing studies from the UK Biobank available at https://pheweb.org/UKB-SAIGE/. The summary statistics from each GWAS for Alzheimer's disease can be found at https://ctg.cncr.nl/software/summary_statistics, https://www.niagads.org/datasets, and https://www.ebi.ac.uk/gwas/. Specifically, 1. The genome-wide survival association study performed by Huang et al. 2017 (NIAGADS ID: NG00058); 2. The genome-wide meta-analysis by

Jansen et al. 201944 (available through: https://ctg.cncr.nl/software/summary_statistics); 3. The genome-wide meta-analysis by Kunkle et al. 2019 (NIAGADS ID: NG00075); 4. The genome-wide meta-analysis by Schwartzentruber et al. 2021 (GWAS catalog ID: GCST90012877); 5. In-house genome-wide associations study imputed using the TOPMed reference panels (see Supplementary Table 3); 6-7. Two whole-exome sequencing analyses of data from ADSP by Bis et al. 2020 (NIAGADS ID: NG00065), and Le Guen et al. 2021 (NIAGADS ID: NG000112); 8. In-house whole-exome sequencing analysis of ADSP (NIAGADS ID: NG00067.v5); 9. In-house whole-genome sequencing analysis of ADSP (NIAGADS ID: NG00067.v5). The single cell RNASeq data for the candidate genes is available in the GEO database under accession code GSE163577 [https://www.ncbi.nlm.nih.gov/geo/query/acc.cgi?acc=GSE163577]. The results of our analysis of UK Biobank and AD genetics can be downloaded at: zihuaihelab.github.io.

Human research participants

Policy information about studies involving human research participants and Sex and Gender in Research.

Reporting on sex and gender	NA
Population characteristics	NA
Recruitment	NA
Ethics oversight	NA

Note that full information on the approval of the study protocol must also be provided in the manuscript.

Field-specific reporting

Please select the one below that is the best fit for your research. If you are not sure, read the appropriate sections before making your selection.

☒ Life sciences ☐ Behavioural & social sciences ☐ Ecological, evolutionary & environmental sciences

For a reference copy of the document with all sections, see nature.com/documents/nr-reporting-summary-flat.pdf

Life sciences study design

All studies must disclose on these points even when the disclosure is negative.

Sample size	The paper focuses on method development, with applications to summary statistics that are publicly available. No individual level datasets were used.
Data exclusions	No data were excluded from the analysis.
Replication	NA
Randomization	NA
Blinding	NA

Reporting for specific materials, systems and methods

We require information from authors about some types of materials, experimental systems and methods used in many studies. Here, indicate whether each material, system or method listed is relevant to your study. If you are not sure if a list item applies to your research, read the appropriate section before selecting a response.

Materials & experimental systems

n/a	Involved in the study	
☒	☐	Antibodies
☒	☐	Eukaryotic cell lines
☒	☐	Palaeontology and archaeology
☒	☐	Animals and other organisms
☒	☐	Clinical data
☒	☐	Dual use research of concern

Methods

n/a	Involved in the study	
☒	☐	ChIP-seq
☒	☐	Flow cytometry
☒	☐	MRI-based neuroimaging
